# Systematic Proteomic Identification of the Heat Shock Proteins (Hsp) that Interact with Estrogen Receptor Alpha (ERα) and Biochemical Characterization of the ERα-Hsp70 Interaction

**DOI:** 10.1371/journal.pone.0160312

**Published:** 2016-08-02

**Authors:** Ahmed E. Dhamad, Zhenqi Zhou, Jianhong Zhou, Yuchun Du

**Affiliations:** Department of Biological Sciences, University of Arkansas, Fayetteville, Arkansas, United States of America; University of Geneva, SWITZERLAND

## Abstract

Heat shock proteins (Hsps) are known to associate with estrogen receptors (ER) and regulate ER-mediated cell proliferation. Historically, the studies in this area have focused on Hsp90. However, some critical aspects of the Hsp-ERα interactions remain unclear. For example, we do not know which Hsps are the major or minor ERα interactants and whether or not different Hsp isoforms associate equally with ERα. In the present study, through a quantitative proteomic method we found that 21 Hsps and 3 Hsp cochaperones were associated with ERα in human 293T cells that were cultured in a medium containing necessary elements for cell proliferation. Four Hsp70s (Hsp70-1, Hsc70, Grp75, and Grp78) were the most abundant Hsps identified to associate with ERα, followed by two Hsp90s (Hsp90α and Hsp90β) and three Hsp110s (Hsp105, HspA4, and HspA4L). Hsp90α was found to be 2–3 times more abundant than Hsp90β in the ERα-containing complexes. Among the reported Hsp cochaperones, we detected prostaglandin E synthase 3 (p23), peptidyl-prolyl cis-trans isomerase FKBP5 (FKBP51), and E3 ubiquitin-protein ligase CHIP (CHIP). Studies with the two most abundant ERα-associated Hsps, Hsp70-1 and Hsc70, using human breast cancer MCF7 cells demonstrate that the two Hsps interacted with ERα in both the cytoplasm and nucleus when the cells were cultured in a medium supplemented with fetal bovine serum and phenol red. Interestingly, the ERα-Hsp70-1/Hsc70 interactions were detected only in the cytoplasm but not in the nucleus under hormone starvation conditions, and stimulation of the starved cells with 17β-estradiol (E2) did not change this. In addition, E2-treatment weakened the ERα-Hsc70 interaction but had no effect on the ERα-Hsp70-1 interaction. Further studies showed that significant portions of Hsp70-1 and Hsc70 were associated with transcriptionally active chromatin and inactive chromatin, and the two Hsps interacted with ERα in both forms of the chromatins in MCF7 cells.

## Introduction

Estrogen receptor alpha (ERα) is a nuclear transcription factor that controls the expression of estrogen responsive genes. Like other members of steroid receptor (SR) superfamily including androgen receptor, progesterone receptor, glucocorticoid receptor and mineralocorticoid receptor, the responsiveness of ERα to its ligands such as 17β-estradiol (E2) is regulated by heat shock proteins (Hsps) and their cochaperones [1–3]. In the absence of estrogenic ligands, ERα is assembled into an Hsp90-based chaperone protein complex, which keeps ERα in a ligand-binding competent but inactive state and prevents it from binding to estrogen-response elements [4–7]. Unliganded ERα is a short-lived protein with a half-life of 4–5 h and is constantly degraded [8]. The degradation is mediated by E3 ubiquitin-protein ligase CHIP (CHIP) and through the ubiquitin-proteasome pathway [9–11]. Upon binding of its ligands, ERα dissociates from Hsp90, dimerizes, binds to the estrogen-response elements, and induces transcription of its target genes through recruiting co-activators [12, 13]. Hsp90 is essential for ERα hormone binding [6], dimer formation [12], and binding to the estrogen-response elements [14].

The Hsps are highly conserved chaperones and play important roles in protein folding, assembly, trafficking and disposition, and stress responses [15, 16]. Human Hsps are classified into six families, Hsp110 (HspH), Hsp90 (HspC), Hsp70 (HspA), Hsp40 (DNAJ), small Hsps (HspB), and chaperonin (HspD/E and CCT) [17, 18]. Hsps vary substantially from one to another with regards to function, expression, and subcellular localization. Some Hsps are constitutively expressed such as Hsc70 and Hsp90β, whereas others are induced by stresses such as Hsp70-1 and Hsp90α [19, 20]. While some Hsps are localized in specific cellular compartments, such as Grp75 in the mitochondria and Grp78 in the endoplasmic reticulum, most Hsps are localized in the cytoplasm and the nucleus [21, 22]. Hsp70 and Hsp90 are among the most abundant cellular proteins, with each family accounting for 1–2% of total cellular protein under normal conditions and 2–4% under stress conditions [23–26]. Despite the fact that Hsp70 and Hsp90 are among the main conserved protective systems in cells [27], they are substantially overexpressed in cancer cells, and the upregulations correlate with poor prognosis [28, 29]. Because of the important roles of Hsp70 and Hsp90 in regulating SRs, and the “addiction” of cancer cells to higher levels of Hsps, inhibitors of Hsp70 and Hsp90 are actively being pursued for treating cancers [23, 24, 28, 30–32].

The extensive studies on the interactions of Hsps with SRs including ERα over the past five decades have established the fundamental roles of Hsps, Hsp90 in particular, in regulating SRs [33]. However, some details are missing and in some cases results are controversial. For examples, because Hsp90α and Hsp90β share 86% sequence [34], it is expected that the two isoforms have similar functions in cells. Probably because of this reason, many publications on studying the roles of Hsp90 in regulating SRs even did not mention which isoforms they used. However, while Hsp90α-knockout mice are viable, Hsp90β-knockout mice are lethal [35, 36]. As myoblasts differentiate into myotubes, Hsp90α disappears and only Hsp90β remains, and the isoform switch is essential for the differentiation [37]. These results suggest that there are critical differences between the two isoforms. Through a quantitative proteomic approach, we have comprehensively identified cellular proteins that are associated with ERα in human 293T cells that were grown in a “complete” culture medium [a medium that was supplemented with growth stimulating factors including phenol red and fetal bovine serum (FBS)]. Here we present the results revealing the interactions between ERα and Hsps/cochaperones at the proteome level. Our proteomic data demonstrate that four Hsp70 family members, Hsp70-1, Hsc70, Grp75 and Grp78, were the predominant Hsps that were associated with ERα in 293T cells, followed by two Hsp90 family members, Hsp90α and Hsp90β, and three Hsp110 family members, Hsp105, HspA4 and HspA4L. In addition, three Hsp cochaperones, prostaglandin E synthase 3 (p23), peptidyl-prolyl cis-trans isomerase FKBP5 (FKBP51) and CHIP, were also identified to associate with ERα. Studies with the two most abundant ERα-associated Hsps, Hsp70-1 and Hsc70, suggest that these two Hsps interact with ERα in the cytoplasm and the nucleus when human breast cancer MCF7 cells were cultured in the conventional laboratory conditions. However, under hormone starvation, the ERα-Hsp70-1/Hsc70 interactions were observed only in the cytosol, and E2 stimulation did not change the pattern. The E2-treatment weakened the ERα-Hsc70 interaction but had no effect on the ERα-Hsp70-1 interaction. Different from Hsp90α, significant portions of Hsp70-1 and Hsc70 were found to be associated with transcriptionally active chromatin and inactive chromatin, and the two Hsps interacted with ERα in both forms of the chromatins in MCF7 cells.

## Materials and Methods

### Cell culture, proteome labeling, and affinity purification

We used the SILAC/AACT (stable isotope labeling with amino acids in cell culture/amino acid-coded tagging) approach to label the proteome of cells [38, 39]. A population of human embryonic kidney 293T cells (American Type Culture Collection, Manassas, VA) were cultured in labeled (Arg-^13^C_6_ and Lys-^13^C_6_^15^N_2_) Dulbecco's Modified Eagle's Medium (DMEM, Thermo Fisher Scientific, Waltham, MA) with 10% dialyzed FBS and 1% penicillin and streptomycin for two weeks and then transiently transfected with a plasmid expressing Flag tag alone. A second population of 293T cells were cultured in unlabeled DMEM with 10% FBS and 1% penicillin and streptomycin, and transiently transfected with a plasmid expressing Flag-ERα. The two population of cells were harvested 48 h after transfection, washed with cold PBS, and incubated in 5 packed cell pellet volumes of lysis buffer I [20 mM Tris-HCl, pH 7.5, 0.5% NP-40, 1 mM EDTA, 10 nM E2, protease inhibitors (Roche, Indianapolis, IN), and phosphatase inhibitors (1 mM Na3VO4, 10 mM NaF, and 10 mM β-glycerophosphate)] on ice for 30 min. The cells were then lysed by douncing with a 15-mL glass dounce homogenizer with a tight-fitting type B pestle (Kontes Glass Co., Vineland, NJ). After adding NaCl and glycerol to final concentrations of 125 mM and 10%, respectively, the extracts were centrifuged at 20,000 g for 15 min at 4°C. The resulting pellets were resuspended in lysis buffer I supplemented with 125 mM NaCl and 10% glycerol and extracted again with sonication (Branson Digital Sonifier 450, Branson Ultrasonics Co., CT) [40]. Protein concentration of the combined and cleared supernatant was determined, and equal amounts of the labeled and unlabeled cell extracts were separately incubated with pre-washed Flag M2 resin (Sigma-Aldrich, St. Louis, MO) for 5 h at 4°C with end-to-end rotation. The beads were then washed extensively with lysis buffer I supplemented with 125 mM NaCl and 10% glycerol. The bound proteins were eluted with elution buffer (10 mM Tris-HCl, pH 7.5, 350 mM NaCl, 1 mM EDTA, 250 mM 3X Flag peptides, and protease inhibitors). The eluates of the two affinity purifications were mixed and fractionated with a 12% SDS-PAGE gel for liquid chromatography-tandem mass spectrometry (LC-MS/MS) analysis. Human breast cancer MCF7 cells (American Type Culture Collection, Manassas, VA) were maintained in Minimum Essential Medium α (MEM α; Thermo Fisher Scientific, Waltham, MA) with 5% FBS and 1% penicillin and streptomycin.

### LC-MS/MS, database search, and data analysis

In-gel digestion, LC-MS/MS analysis, and protein identification/quantification with the Maxquant (version 1.0.13.13) and Mascot (version 2.2; Matrix Science, Boston, MA) by searching against a composite target-decoy International Protein Index (IPI) human protein database (version 3.52) were performed as described previously [41]. In this SILAC/AACT approach, because the Flag-ERα expressing cells and the Flag expressing cells were cultured in the unlabeled medium and stable-isotope-labeled medium, respectively, and the eluates from the two affinity purifications of equal amounts of the unlabeled cell extract and labeled cell extract were mixed and analyzed by LC-MS/MS, the relative intensities of the paired isotopic peaks of peptides (i.e., light/heavy ratios: L/H ratios) reflect the binding profile of the protein to ERα. Whereas the L/H ratios for the nonspecific binding proteins were around 1, the ratios for the proteins that specifically bind to ERα were significantly larger than 1 due to affinity enrichment of the proteins [42, 43]. Search results were further processed by Scaffold software (version 4.4.7; Proteome Software Inc., Portland, OR) for viewing protein and peptide identification information. In the Scaffold analysis, protein identification probability with at least two peptides was set to 99% and the peptide identification probability was set to 95%. The normalized spectral abundance factors (NcSAFs) were calculated as described [44, 45]. The normalization was applied only to the identified Hsps and cochaperones to estimate the relative level of each protein within the identified Hsps and cochaperones that were associated with ERα [44, 45]. Spectral counts for peptides shared among the identified Hsps were counted only once, and distributed based on the number of unique spectral counts to each isoform [46].

### The E2 treatment and subcellular fractionation

The MCF7 cells were cultured in the phenol-red free MEM α supplemented with 5% charcoal-treated FBS (Hyclone, Logan, UT) for 3–4 days and then treated with either 100 nM E2 or ethanol (control) for 24 h. The cells were then harvested, washed twice with cold PBS, resuspended in 5 packed cell pellet volumes of hypotonic buffer (10 mM Tris-HCl, pH 8.0, 5 mM KCl, and 1.5 mM MgCl_2_, and protease inhibitors) supplemented with 100 nM E2 for the E2-treated cells or ethanol for the control cells. The cells were incubated on ice for 20 min. After adding phosphatase inhibitors (1 mM Na3VO4, 10 mM NaF, and 10 mM glycerophosphate) to the cell suspension, the cells were lysed by douncing 12 times with a 15-mL glass dounce homogenizer with a tight-fitting type B pestle. After centrifugation at 500 g for 10 min at 4°C, the pellet was saved and the supernatant was cleared by centrifugation at 10,000 g for 15 min at 4°C. The cleared supernatant was supplemented with 15 mM Tris-HCl, pH 8.0, 140 mM NaCl, 1% Triton X-100, 0.1% SDS and 3 mM EDTA, and saved as cytosolic fraction. The pellet from the 500 g centrifugation was resuspended in hypotonic buffer and dounced 5 times. After centrifugation at 500 g for 10 min at 4°C, the pellet was washed twice with hypotonic buffer and saved as nuclei. The isolated nuclei were resuspended in lysis buffer II (25 mM Tris-HCl, pH 8.0, 140 mM NaCl, 1% Triton X-100, 3 mM EDTA, 0.1% SDS, protease inhibitors, and phosphate inhibitors) supplemented with 100 nM E2 for the E2-treated samples or ethanol for the control samples. The nuclei were then sonicated on ice, centrifuged at 10,000 g for 15 min at 4°C, and the resulting supernatant was designated as nuclear fraction.

### Cross-linking, immunoprecipitation (IP), and Western blotting

In-cell cross-linking was performed using the cell-permeable cross-linking reagent dithiobis (succinimidylpropionate) (DSP) (Thermo Fisher Scientific, Waltham, MA). The MCF7 cells in plates were washed twice with PBS at room temperature and incubated with 1 mM DSP in DMEM at 37°C for 15 min. After removal of the cross-linker solution, the cells were incubated with quenching solution (100 mM Tris-HCl, pH 8.0 in DMEM) at 37°C for 10 min. Quenching solution was removed, and the cells were washed twice with PBS and lysed for IPs. The IPs and Western blotting were performed as described previously [47, 48]. Antibodies used in this study were purchased from the following commercial sources: Anti-ERα, p300, and NCoR antibodies from Santa Cruz Biotech (Santa Cruz, Dallas, TX; catalog no.: Anti-ERα, sc-8002; anti-p300, sc-584; anti-NCoR, sc-1609), anti-Hsp70-1 and Hsc70 antibodies from Enzo life science (Farmingdale, NY; catalog no.: anti-Hsp70-1, ADI-SPA-810; anti-Hsc70, ADI-SPA-815), anti-Hsp90α from Epitomics (Burlingame, CA; catalog no., 3670–1)), anti-histone H3 from Cell signaling (Danvers, MA; catalog no., 9715), and anti-tubulin from Sigma-Aldrich (St. Louis, MO; catalog no., T9026). Quantification of protein bands in Western blotting was performed using ImageJ software.

### Extraction of chromatin-binding protein, and transcriptionally active chromatin and inactive chromatin

Chromatin-binding protein was extracted with 0.3% SDS and 250 units/mL benzonase as described by Yang *et*. *al*. (2014) [49]. Briefly, after MCF7 cells were resuspended in a radioimmunoprecipitation assay (RIPA) buffer (50 mM Tris-HCl, pH 7.4, 150 mM NaCl, 0.25% deoxycholic acid, 1% NP-40, and 1 mM EDTA) supplemented with 200 μM phenylmethylsulfonyl fluoride (PMSF), 1 mM sodium orthovanadate and protease inhibitors, the cells were homogenized by passing through a 22G needle 10 times, followed by an incubation on ice for 20 min. The chromatin was separated from the soluble protein (S) by a centrifugation at 1,000 g, and the isolated chromatin was extracted with 0.3% SDS and 250 units/mL benzonase (EMD Millipore, Billerica, MA) on ice for 10 min. The digested chromatin was centrifuged at 1,000 g, and the resulting supernatant was designated as chromatin-binding protein (CB). Transcriptionally active chromatin and inactive chromatin were extracted with different concentrations of salt according to Henikoff *et*.*al*. (2009) and Yang *et*. *al*. (2014) [49, 50]. Briefly, after MCF7 cells were lysed with a lysis buffer (10 mM Tris-HCl, pH 8.0, 10 mM KCl, 1.5 mM MgCl_2_, 340 mM sucrose, 10% glycerol, 1 mM DTT, 0.1% Triton X-100, and protease inhibitors) on ice for 8 min, cytoplasmic protein (C) was separated from the nuclei with a 1,300 g centrifugation. The washed nuclei were digested with 2,000 gel units/mL micrococcal nuclease (New England Biolabs, Ipswich, MA) in the lysis buffer described above plus 1 mM CaCl_2_ at 37°C for 10 min, and the reaction was stopped by 2 mM EGTA. After centrifugation at 1,300 g for 10 min at 4°C, the supernatant (nuclear soluble protein: NS) was removed and the digested nuclei were washed and first treated with 150 mM NaCl at 4°C for 2 h for extracting active chromatin (Ch1) and then with 600 mM NaCl at 4°C overnight for extracting inactive chromatin (Ch2).

### Statistical analysis

Statistical analysis was performed using one-way ANOVA (PSI-PLOT, Pearl River, NY). A *p*-value of <0.05 was considered significant.

## Results

### Identification of Hsps and their cochaperones that associate with ERα

We used a SILAC/AACT-based quantitative proteomic method to systematically identify cellular proteins that were associated with ERα [42, 43]. Through this approach, a subset of Hsps and their cochaperones were identified to associate with ERα (Table 1). Most of the Hsps and cochaperones were identified with high confidence with LC-MS/MS (S1 Table), which can be reflected by the very low PEP (posterior error probability) values for the identifications (Table 1).

**Table 1:** Heat shock proteins and their cochaperones that were identified to associate with ERα in human cells.

Family	Gene names	Protein names (short names)	UniProt ID	Unique peptides	Unique spectra	Sequence coverage (%)*	NcSAF	L/H ratios†	PEP‡
Hsp70	*HSPA1A*	Heat shock 70 kDa protein 1A/1B (Hsp70-1)	P08107	28	927	60.5	0.286	8.9	0
	*HSPA2*	Heat shock-related 70 kDa protein 2	P54652	12	38	37.6	0.012	8.0	3.2E-198
	*HSPA5*	78 kDa glucose-regulated protein (Grp78)	P11021	35	266	51.4	0.080	10.7	0
	*HSPA6*	Heat shock 70 kDa protein 6	P17066	6	15	22.9	0.005	8.0	1.1E-121
	*HSPA8*	Heat shock cognate 71 kDa protein (Hsc70)	P11142	25	810	60.5	0.248	11.7	0
	*HSPA9*	Stress-70 protein, mitochondrial (Grp75)	P38646	33	425	49.9	0.124	12.1	0
Hsp90	*HSP90AA1*	Heat shock protein Hsp90-alpha (Hsp90α)	P07900	18	221	45.4	0.057	6.7	3.3E-195
	*HSP90AB1*	Heat shock protein Hsp90-beta (Hsp90β)	P08238	19	72	47.1	0.022	5.5	1.7E-144
Hsp110	*HSPH1*	Heat shock protein 105 kDa (Hsp105)	Q92598	25	74	34.1	0.016	24.6	3.7E-241
	*HSPA4*	Heat shock 70 kDa protein 4 (HspA4)	P34932	38	154	54.0	0.032	4.7	0
	*HSPA4L*	Heat shock 70 kDa protein 4L (HspA4L)	O95757	22	60	35.9	0.012	26.2	9.2E-163
Hsp40	*DNAJA2*	DnaJ homolog subfamily A member 2	O60884	2	4	6.1	0.002	2.1	9.0E-22
	*DNAJA3*	DnaJ homolog subfamily A member 3, mitochondrial	Q96EY1	5	16	16.9	0.005	12.7	1.0E-55
	*DNAJB1*	DnaJ homolog subfamily B member 1	P25685	4	6	12.6	0.003	5.6	1.7E-15
	*DNAJB4*	DnaJ homolog subfamily B member 4	Q9UDY4	2	4	5.9	0.002	4.7	2.6E-07
	*DNAJB6*	DnaJ homolog subfamily B member 6	O75190	3	8	9.2	0.004	2.3	1.2E-12
	*DNAJC7*	DnaJ homolog subfamily C member 7	Q99615	4	8	9.9	0.003	2.0	4.9E-25
	*DNAJC9*	DnaJ homolog subfamily C member 9	Q8WXX5	10	21	35.0	0.013	14.7	0.068
	*DNAJC10*	DnaJ homolog subfamily C member 10	Q8IXB1	2	6	2.8	0.001	5.9	1.2E-07
Small Hsps	*HSPB8*	Heat shock protein beta-8 (Hsp22)	Q9UJY1	2	4	9.7	0.003	12.0	0.0035
Chaperonin	*HSPE1*	10 kDa heat shock protein, mitochondrial	P61604	3	10	31.4	0.016	15.6	8.3E-27
Cochaperones	*STUB1*	E3 ubiquitin-protein ligase CHIP (CHIP)	Q9UNE7	14	57	45.5	0.030	21.6	1.5E-111
	*FKBP5*	Peptidyl-prolyl cis-trans isomerase FKBP5 (FKBP51)	Q13451	18	39	38.5	0.014	16.5	8.0E-108
	*PTGES3*	Prostaglandin E synthase 3 (p23)	Q15185	4	12	15.6	0.012	171.1	2.0E-08

* Coverage of all peptide sequences matched to the identified protein sequence (%).

^†^ Ratios of light peptides (derived from Flag-ERα-expressing cells) versus heavy peptides (derived from Flag alone-expressing cells).

^‡^PEP: posterior error probability.

To examine the abundance of the identified Hsps and cochaperones that were associated with ERα, we calculated NcSAF for each protein [51]. NcSAF is based on spectral counting for each protein in LC-MS/MS analysis, and a larger NcSAF value reflects the higher abundance of the protein in biological samples [44, 46, 51, 52]. The most abundant Hsps that were associated with ERα were four Hsp70 family members, Hsp70-1, Hsc70, Grp75 and Grp78, with the NcSAF values in the range of 0.08–0.286. The L/H ratios for all the identified Hsp70s varied in a narrow range of from 8 to 12, suggesting they were enriched by affinity purification similarly.

Two Hsp90 family members and three Hsp110 family members were also identified to be abundant in the ERα-containing complexes, though at significantly less levels than the four Hsp70 family members described above (Table 1). Among the 5 reported Hsp90 members [17], Hsp90α and Hsp90β, which share 86% sequence homology [34], were identified to associate with ERα. The NcSAF values for Hsp90α and Hsp90β were 0.057 and 0.022, respectively, and thus the former was 2.6-fold of that of the latter, suggesting that Hsp90α is 2–3 times more abundant than Hsp90β in the ERα-containing protein complexes. It is known that while the expression of Hsp90α is inducible, Hsp90β is constitutively expressed [53]. The L/H ratios were similar for Hsp90α and Hsp90β (6.7 and 5.5, respectively), suggesting the proportions of those that were specifically associated with ERα to those of non-specific bindings for the two isoforms were similar. The Hsp110 members are known as nucleotide exchanger factors (NEFs) of Hsp70 and interact with Hsc70 [17, 54]. Three Hsp110 members, Hsp105, HspA4 and HspA4L, were identified to abundantly associate with ERα (Table 1). The abundances of the three Hsp110 members were comparable to those of Hsp90α and Hsp90β, with the NcSAF values in the range of 0.012–0.032. HspA4 and HspA4L were originally considered as members of Hsp70 [55], but now are classified as members of the Hsp110 family [56]. It is noteworthy that Hsp105 and HspA4L were identified with high L/H ratios, suggesting that they were highly enriched by anti-Flag antibody.

The Hsp40 (DNAJ proteins) constitutes the largest subgroup of the Hsp family, up to 50 members, in human cells. One of the major functions of Hsp40 is to couple with Hsp70 to facilitate folding of Hsp70 client proteins [27]. We identified eight Hsp40 members in this study, and all of them were identified with smaller NcSAF values compared with other identified Hsps except for DNAJC9, which was identified with a NcSAF value comparable to those for the Hsp110 members. These results suggest that the majority of Hsp40 members are not abundant in the ERα-containing complexes. Based on the fact that Hsp40 physically interacts with Hsp70 [27], it is likely that Hsp40 interacts with ERα indirectly and the interactions are mediated by Hsp70.

Multiple Hsp cochaperones, including p23, FKBP51, FKBP52, protein phosphatase 5 (PP5) and cyclophilin 40 (Cyp40), have been reported to couple with Hsp90 to facilitate the function of SRs [1, 33]. Most of these cochaperones contain tetratricopeptide repeat domains, which bind to the EEDV motif of Hsp90/Hsp70 [57], and are typically assembled into SR complexes at the final stages of assembly to form the mature, hormone-competent states of SRs [58, 59]. Among the reported cochaperones, we identified p23, FKBP51, and CHIP but were not able to detect FKBP52, Cyp40, and PP5 (Table 1). Notably, CHIP was identified with a larger NcSAF value (0.03), which was comparable to those for the two Hsp90 family members and the three Hsp110 family members, suggesting that CHIP is also abundantly associated with ERα. CHIP has been shown to interact with ERα via its tetratricopeptide repeat domain and mediates ERα degradation through the ubiquitin-proteasome pathway in the nucleus [10, 11].

### Hsp70-1 and Hsc70 interact with ERα in the cytoplasm and the nucleus

**Fig 1:**
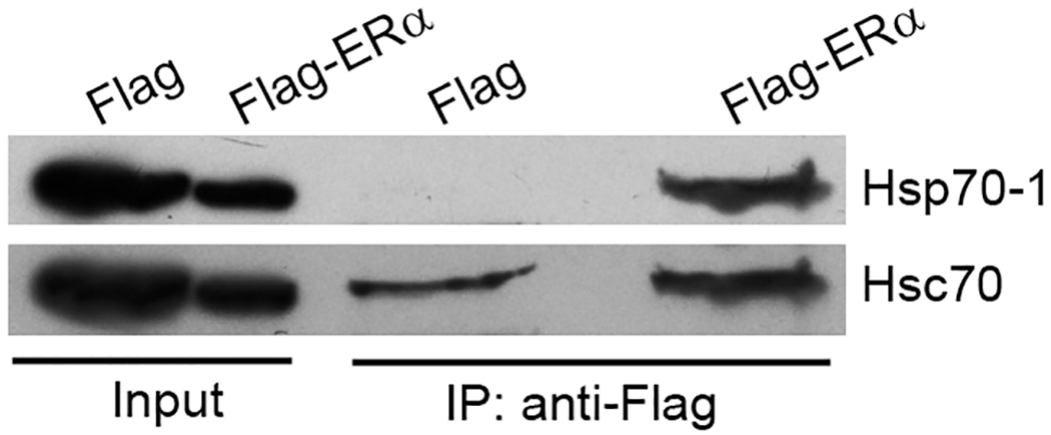
Verification of the interaction between Flag-ERα and endogenous Hsp70-1/Hsc70. The 293T cells were transfected with a plasmid that expresses Flag alone (control) or Flag-ERα. Forty eight hours after transfection, the cells were harvested, lysed, and the resulting total protein was pulled down by immobilized anti-Flag antibody. The bound proteins were analyzed with Western blotting using anti-Hsp70-1 and anti-Hsc70 antibodies.

**Fig 2:**
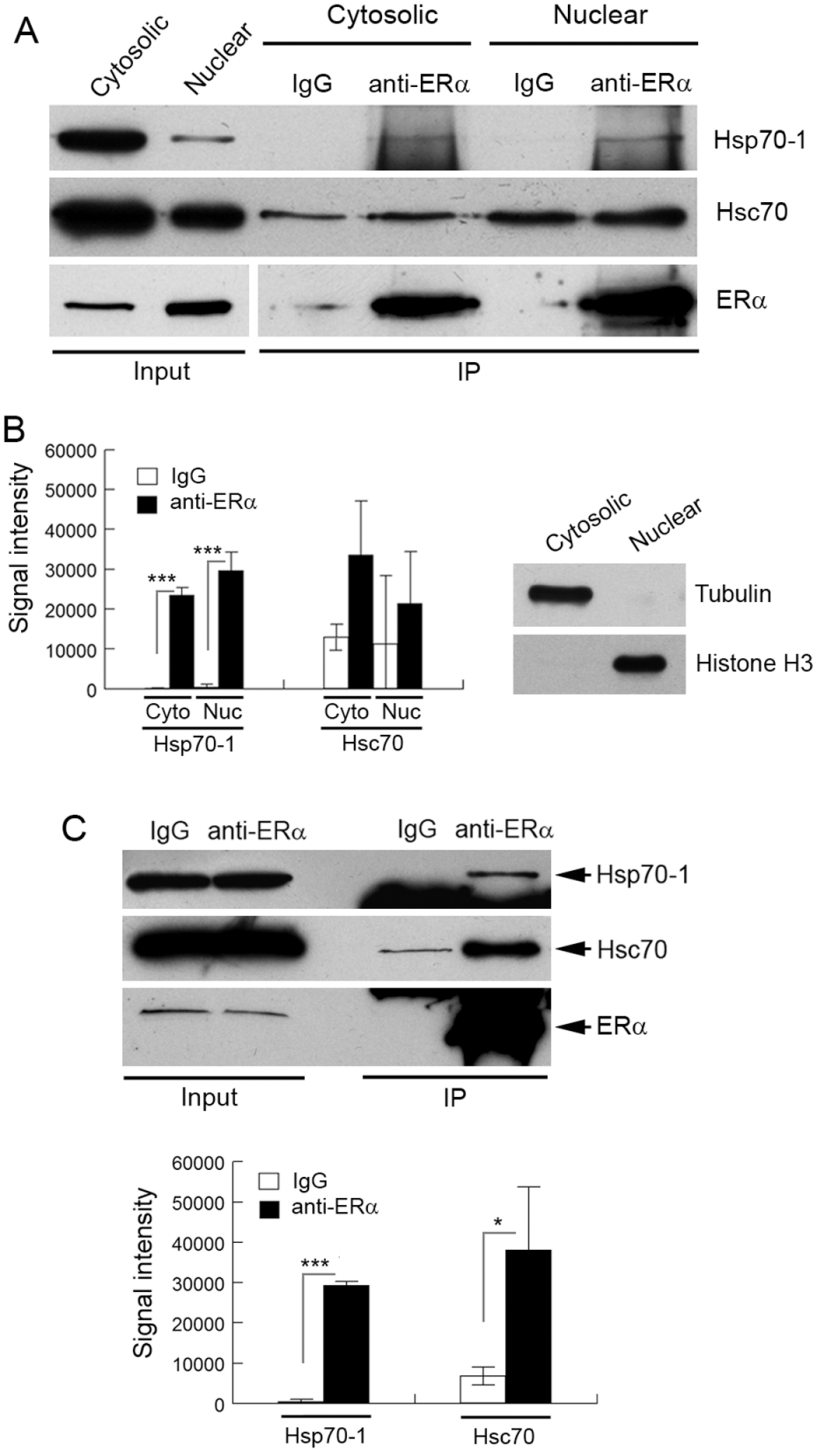
Endogenous ERα interacts with endogenous Hsp70-1 and Hsc70. (A) The cytosolic and nuclear extracts of MCF7 cells were immunoprecipitated by anti-ERα antibody or an isotype-matched, unrelated control IgG, and the immunoprecipitated protein was analyzed by Western blotting with the indicated antibodies. (B) Left panel, quantification of the IP protein bands in Western blots. Signal intensity values were arbitrary numbers obtained by analyzing the protein bands with ImageJ software. Right panel, validation of the cytosolic and nuclear fractionations. Tubulin and histone H3 were used as markers for the cytosolic and nuclear fractions, respectively. (C) The MCF7 cells were treated with the cell-permeable cross-linking reagent DSP and whole cell lysate of the DSP-treated cells was immunoprecipitated by anti-ERα antibody or a control IgG, followed by Western blot analyses with the indicated antibodies. Values in the Western blot quantifications in (B) and (C) were the means ± S.D. of three separate sample preparations. Cyto, cytosolic; Nuc, nuclear. * and *** denote *p* < 0.05 and *p* < 0.001, respectively.

The role of Hsp90 in regulating the assembly, trafficking, and transcriptional activity of ERα has been studied extensively [1]. Compared with Hsp90, much less is known about the role of Hsp70 in regulating ERα and some results are controversial [5, 7, 60]. In this study, we found that Hsp70-1 and Hsc70 were the two most abundant Hsps that were associated with ERα (Table 1). As the first step to characterizing these important interactions, we proceeded to verify the interaction of ERα with Hsp70-1/Hsc70 using IP and Western blotting. Consisting with our proteomic data, the IP results obtained with the 293T cells ectopically expressing Flag-ERα demonstrate that Flag-ERα interacted with endogenous Hsp70-1 and Hsc70 (Fig 1). To examine if endogenous ERα interacts with endogenous Hsp70-1/Hsc70 and determine the subcellular site where the ERα-Hsp70-1/Hsc70 interactions occur in ERα-positive breast cancer cells, we performed IPs using cytosolic and nuclear proteins of human breast cancer MCF7 cells as starting materials, respectively. The results demonstrate that anti-ERα antibody precipitated significantly more Hsp70-1 than the control IgG precipitated in both the cytosolic fractions and the nuclear fractions (Fig 2A, top row; Fig 2B, left panel). However, the amounts of Hsc70 that were precipitated by anti-ERα antibody and the control IgG were not statistically significantly. In addition, we observed large variations on Hsc70 in the IP results among different sample preparations (Fig 2A, middle row; Fig 2B, left panel). The interactions between SRs and Hsps are typically transient and weak by nature [61]. To confirm the interaction of endogenous ERα with Hsc70 and to further validate the specific ERα-Hsp70-1 interaction, we used the cell-permeable cross-linking reagent DSP to treat MCF7 cells and then used whole cell lysate of the DSP-treated cells to perform IPs and Western blotting. The results demonstrate that anti-ERα antibody precipitated significantly more Hsp70-1 and Hsc70 proteins than the IgG precipitated after the cross-linking treatment (Fig 2C). These results suggest that Hsp70-1 and Hsc70 indeed specifically interact with ERα in addition to the nonspecific interactions. We have confirmed the effectiveness of our subcellular fractionation by performing Western blot analysis using markers of the cytoplasm and the nucleus (Fig 2B, right panel).

### Hsp70-1 and Hsc70 interact with ERα in transcriptionally active and inactive chromatins

**Fig 3:**
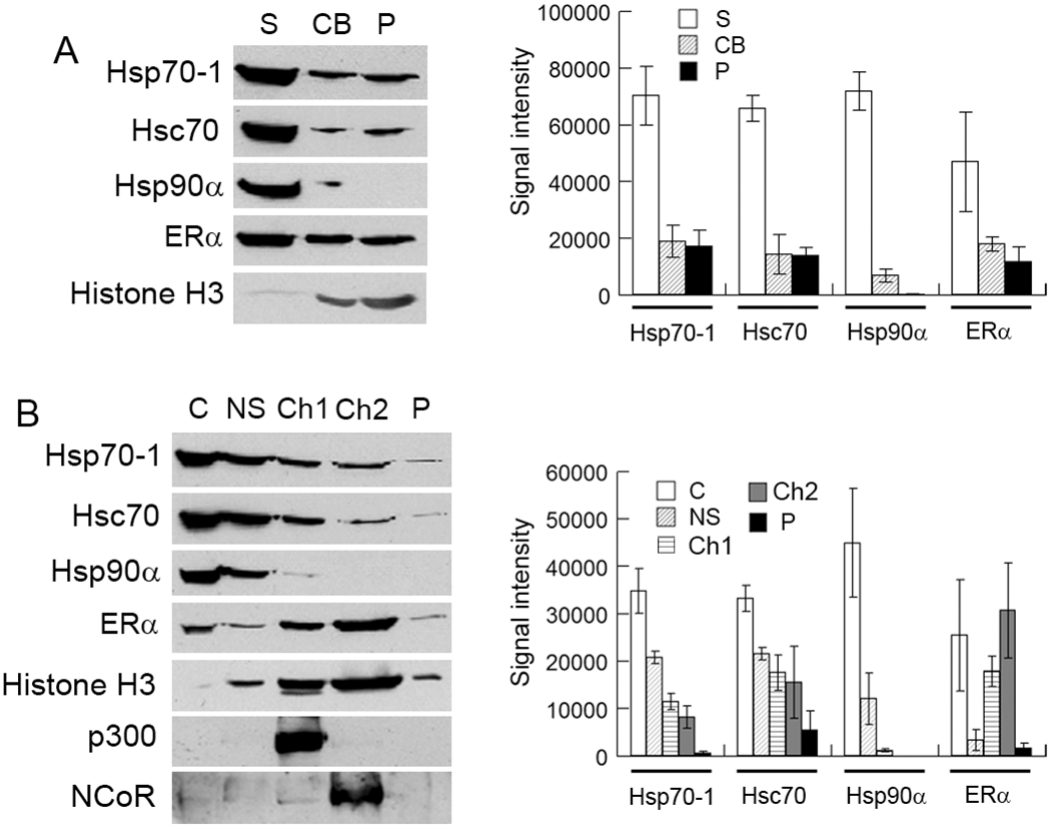
Hsp70-1 and Hsc70 are associated with chromatin. (A) The MCF7 cell extract was fractionated into soluble protein (S), chromatin-binding protein (CB), and the pellet (P), and then analyzed by Western blotting with the indicated antibodies (left panel). Right panel, quantification of Western blots. (B) The MCF7 cell extract was fractionated into cytoplasmic protein (C), nuclear soluble protein (NS), transcriptionally active chromatin (Ch1) and inactive chromatin (Ch2), and analyzed by Western blotting with the indicated antibodies (left panel). Right panel, quantification of Western blots. Histone H3, p300, and NCoR were used as markers of chromatin-binding protein, active chromatin, and inactive chromatins, respectively. Signal intensity values in the Western blot quantifications were arbitrary numbers obtained by analyzing the protein bands with ImageJ software. Values in the Western blot quantifications were the means ± S.D. of three separate sample preparations.

To characterize the interactions of ERα with Hsp70-1/Hsc70, we fractionated MCF7 cell extracts into soluble protein (S), chromatin-binding protein (CB) and the remaining pellet (P), and analyzed those fractions with Western blotting. The results demonstrate that significant portions of Hsp70-1 and Hsc70 were associated with chromatin and the remaining pellets (Fig 3A). In contrast, the amount of Hsp90α associated with chromatin was neglectable and none was detected in the remaining pellet. As expected, a large portion of ERα, a transcriptional factor, was also associated with chromatin and the pellet. The analysis of a marker of chromatin-binding protein, histone H3, confirmed that the method we used for extracting chromatin-binding protein was effective (Fig 3A). To examine how Hsp70-1 and Hsc70 are associated with chromatin, we fractionated MCF7 cell extracts into cytoplasmic protein (C), nuclear soluble protein (NS), transcriptionally active chromatin (Ch1), and inactive chromatin (Ch2) [49]. The results demonstrate that significant portions of Hsp70-1 and Hsc70 were associated with active chromatin and inactive chromatin (Fig 3B). In contrast, only a tiny amount of Hsp90α was associated with active chromatin and none was detected to associate with inactive chromatin. The portions of Hsp70-1, Hsc70, and Hsp90α that existed as nuclear soluble protein were comparable among the three Hsps (Fig 3B). These results suggest that different from Hsp90α, which is localized almost exclusively in the cytoplasm and in the nucleus as non-chromatin-binding protein, Hsp70-1 and Hsc70 are also associated with active chromatin and inactive chromatin in addition to being localized in the cytoplasm and in the nucleus as non-chromatin-binding protein. Strikingly, a large portion of ERα was associated with inactive chromatin when the MCF7 cells were cultured in the “complete” medium. We have verified our active/inactive chromatin extraction protocol with a well-established coactivator–p300 and a corepressor–NcoR, which are typically associated with transcriptionally active chromatin and inactive chromatin, respectively [62, 63] (Fig 3B, left panel).

**Fig 4:**
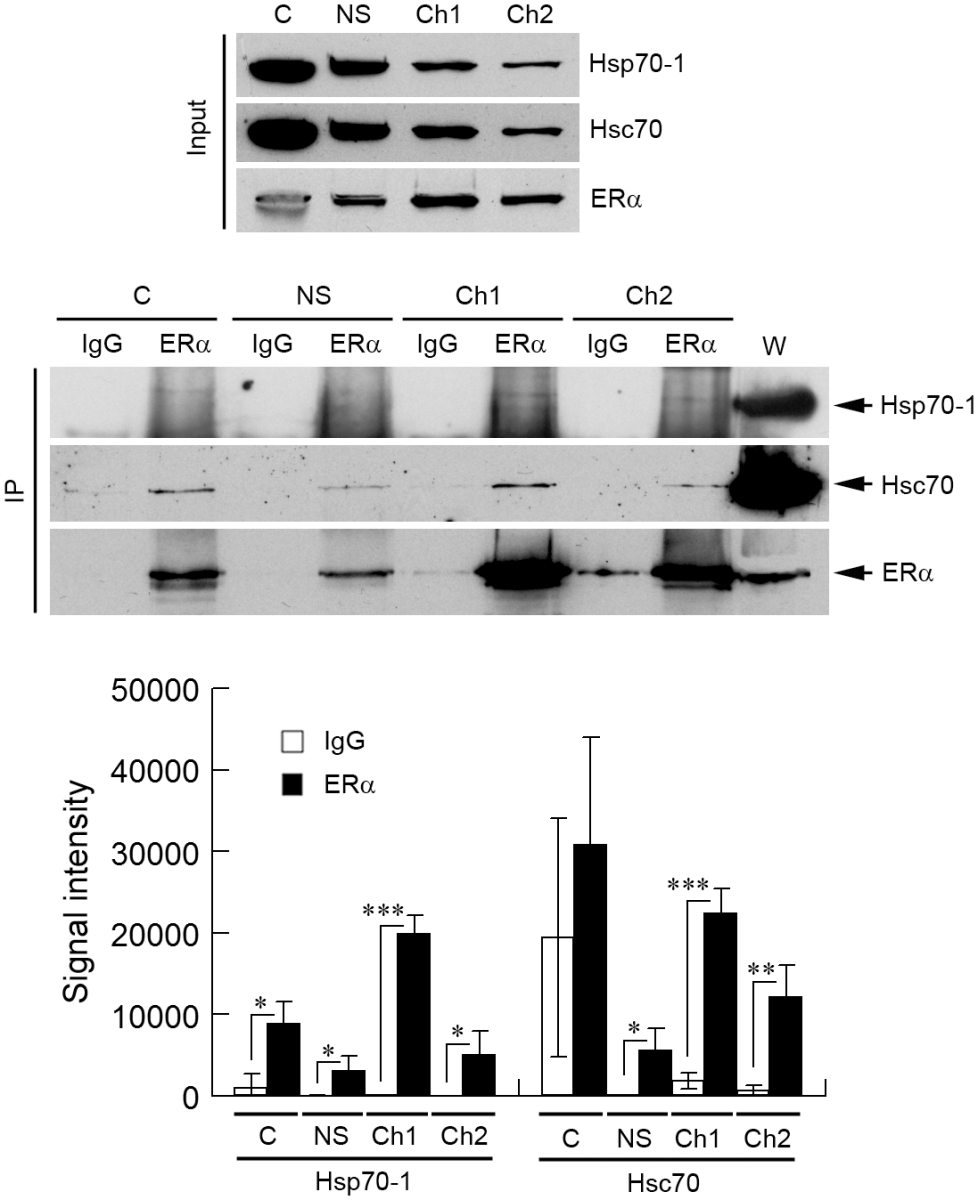
Hsp70-1 and Hsc70 interact with ERα in chromatin. Anti-ERα antibody (ERα) and an isotype-matched, unrelated control IgG were used to immunoprecipitate proteins from cytoplasmic (C), nuclear soluble (NS), transcriptionally active chromatin (Ch1), and inactive chromatin (Ch2) fractions prepared from MCF7 cells. The subcellular proteins were prepared as for Fig 3 except that the inactive chromatin (Ch2) was obtained through sonication instead of elution with 600 mM NaCl. Signal intensity values in the Western blot quantifications were arbitrary numbers obtained by analyzing the protein bands with ImageJ software. Values in the Western blot quantifications were the means ± S.D. of three separate sample preparations. W, whole cell lysate. *, **, and *** denote *p* < 0.05, *p* < 0.01, and *p* < 0.001, respectively.

To examine in which subcellular fraction Hsp70-1 and Hsc70 interact with ERα, we performed IPs using fractionated (cytoplasmic, nuclear soluble, active chromatin, and inactive chromatin fractions) proteins from MCF7 as starting materials. The results demonstrate that anti-ERα antibody precipitated significantly more Hsp70-1 and Hsc70 than the control IgG precipitated in all four fractions tested except for Hsc70 in the cytosolic fraction due to large variations among different sample preparations (Fig 4). We have confirmed the presence of ERα in the expected samples by probing the membrane with anti-ERα body (Fig 4, middle panel). It seemed that the precipitated amounts of Hsp70-1 and Hsc70 correlated with the amount of ERα that was precipitated, which in turn seemed to be correlated with the level of ERα in input samples (Fig 4, top and middle panels; S1 Fig). In addition, despite that the majority of Hsp70-1 and Hsc70 were localized in cytoplasm and in the nucleus as soluble protein (Figs 3B and 4, top panel), significant portions of the ERα-Hsp70-1 and ERα-Hsc70 interactions occurred in the active chromatin and inactive chromatin (Fig 4, middle and low panels), suggesting that the levels of Hsp70-1 and Hsc70 do not affect the amounts of the ERα-Hsp70-1 and ERα-Hsc70 interactions. In short, the results in this section demonstrate that Hsp70-1 and Hsc70 interact with ERα in both active chromatin and inactive chromatin.

### ERα interacts with Hsp70-1/Hsc70 in the cytoplasm under conditions of hormone starvation/stimulation

**Fig 5:**
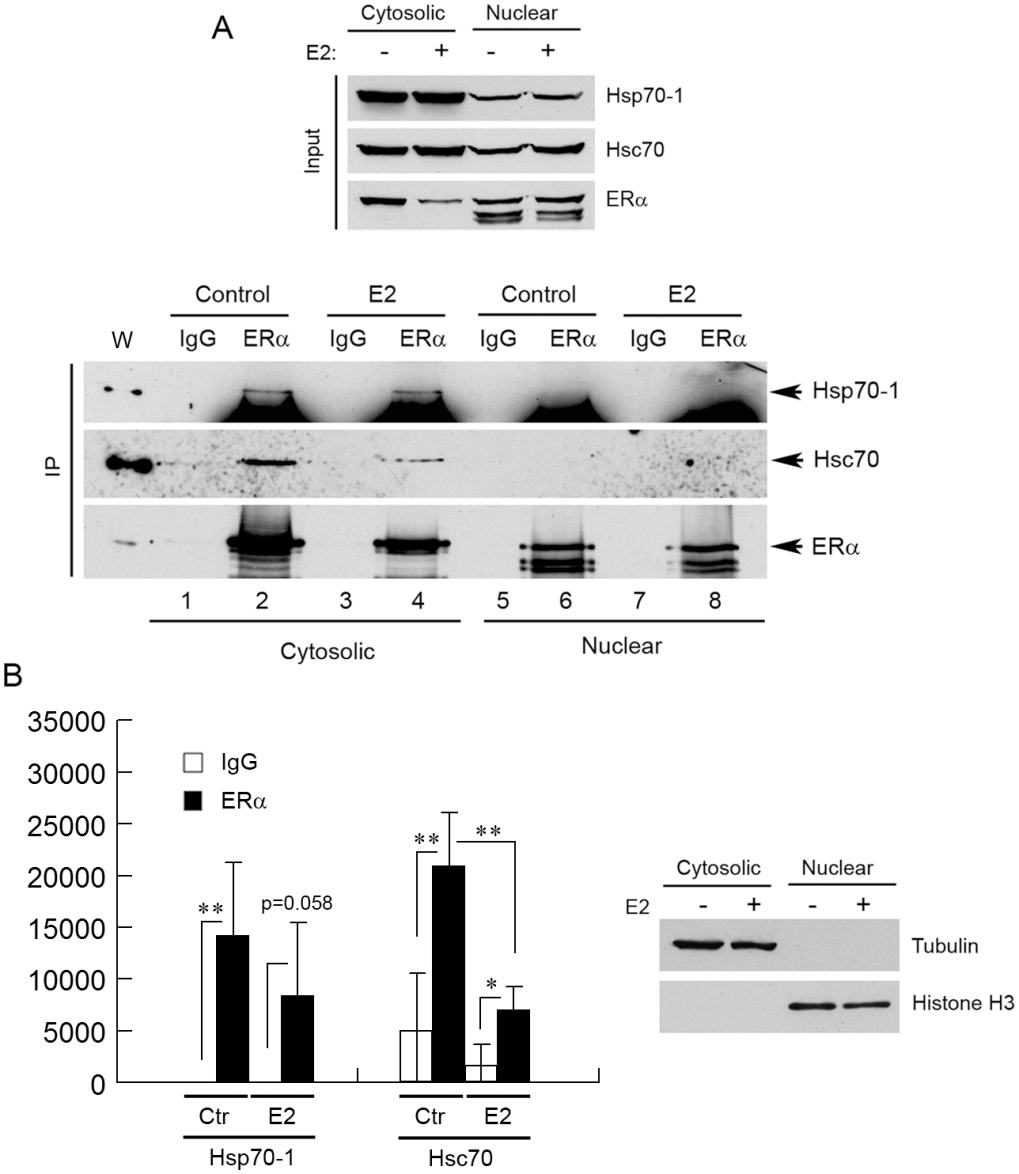
ERα interacts with Hsp70-1 and Hsc70 in the cytoplasm under conditions of hormone starvation/stimulation. (A) The MCF7 cells were cultured under hormone starvation conditions for 3–4 days and then treated with either 100 nM E2 or ethanol (control) for 24 h. The cytosolic and nuclear extracts of the treated cells were then immunoprecipitated by anti-ERα antibody or a control IgG, and the immunoprecipitated protein was analyzed by Western blotting with the indicated antibodies. (B) Left panel, quantification of Western blots. Only the Hsp70-1 and Hsc70 protein bands in the cytosolic fractions were quantified. Signal intensity values in the Western blot quantifications were arbitrary numbers obtained by analyzing the protein bands with ImageJ software. Values in the Western blot quantifications were the means ± S.D. of four separate sample preparations. Right panel, validation of the cytosolic and nuclear fractionations. Tubulin and histone H3 were used as markers for the cytosolic and nuclear fractions, respectively. W, whole cell lysate. Ctr, control. * and ** denote *p* < 0.05 and *p* < 0.01, respectively.

To examine the effect of estrogens on the interaction of ERα with Hsp70-1/Hsc70 in ERα-positive cells, we cultured MCF7 cells in the phenol-red free MEM α supplemented with 5% charcoal-treated FBS for 3–4 days, and then treated the cells with either 100 nM E2 or ethanol (control) for 24 h. We then harvested the cells, fractionated the cell extracts into cytosolic and nuclear fractions, and performed IPs using the cytosolic and nuclear fractions, respectively, as starting materials. The results demonstrate that anti-ERα antibody immunoprecipitated more Hsp70-1 and Hsc70 than the IgG precipitated in the cytosolic fractions (Fig 5A, low panel; compare lane 2 with lane 1, and lane 4 with lane 3; Fig 5B, left panel), suggesting that ERα interacts with Hsp70-1 and Hsc70 in the cytoplasm under conditions of hormone starvation and the subsequent hormone stimulation. The E2 treatment had no significant effect on the ERα-Hsp70-1 interaction, but significantly weakened the interaction between ERα and Hsc70 in the cytoplasm (Fig 5A, low panel; compare lane 4 with lane 2; Fig 5B, left panel). These results are consistent with the previous observations, which showed that Hsp70 was still associated with progesterone receptors in the presence of progesterone but the levels of the association decreased compared with in the absence of progesterone [64, 65]. Anti-ERα antibody did not precipitate any detectable amount of Hsp70-1 and Hsc70 from the nuclear fractions either in the absence or presence of E2 (Fig 5A, low panel; lanes 5–8). Compared with the results shown in Fig 2, which were obtained with the MCF7 cells cultured under conventional laboratory conditions (i.e., a culture medium supplemented with 5% FBS and phenol red), the ERα-Hsp70-1/Hsc70 interactions observed under E2 starvation/stimulation conditions appeared to be different: under the former conditions the interactions were observed in both the cytoplasm and the nucleus (Fig 2) and under the latter conditions in the cytoplasm only (Fig 5). These results suggest that certain factors, potentially not just E2, in the culture media dictate whether ERα interacts with Hsp70-1/Hsc70 in the cytoplasm or the nucleus.

**Fig 6:**
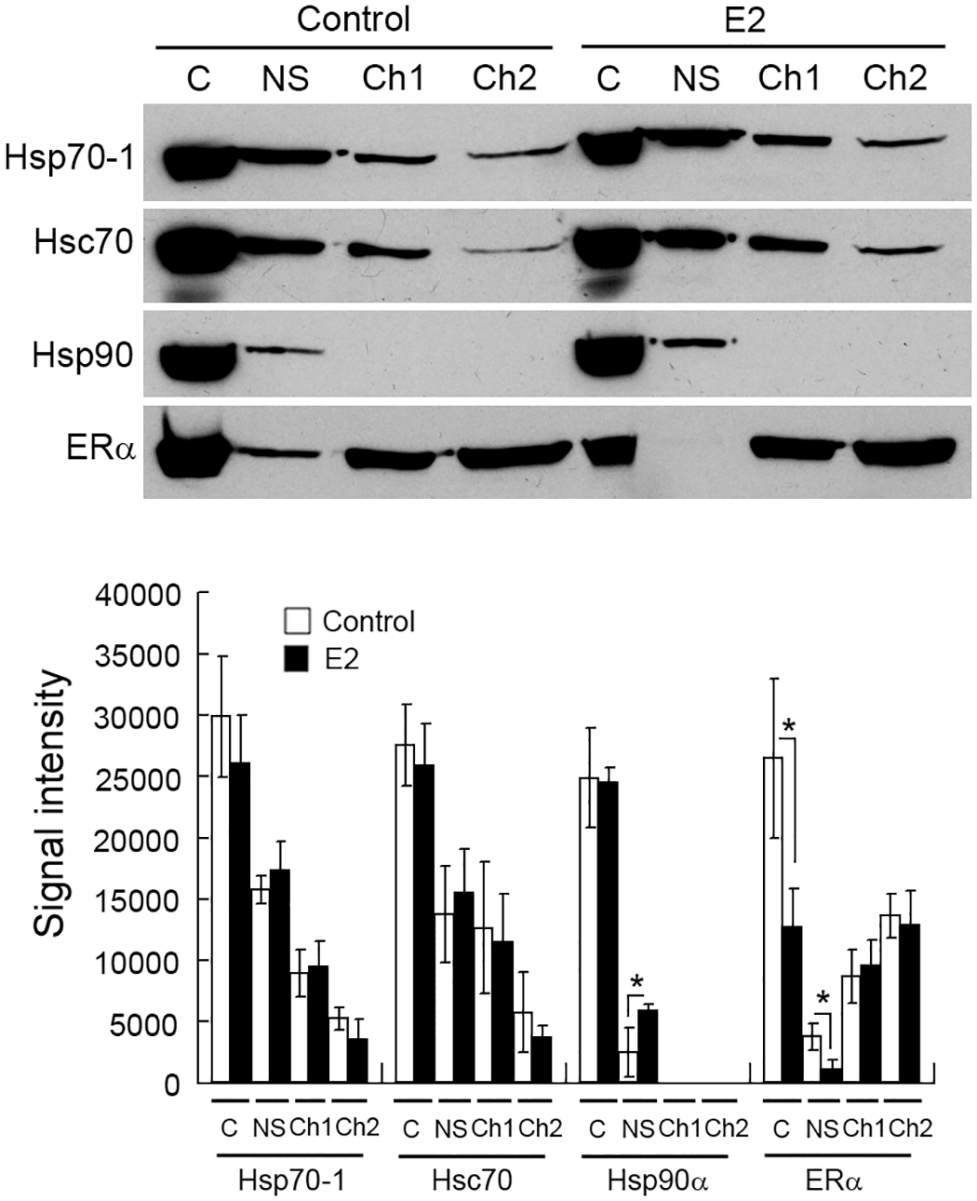
Estradiol does not affect the association of Hsp70-1 and Hsc70 with chromatin. The MCF7 cell extract was fractionated into cytoplasmic protein (C), nuclear soluble protein (NS), transcriptionally active chromatin (Ch1) and inactive chromatin (Ch2), and analyzed by Western blotting with the indicated antibodies (top panel). Signal intensity values in the Western blot quantifications were arbitrary numbers obtained by analyzing the protein bands with ImageJ software. Values in the Western blot quantifications were the means ± S.D. of three separate sample preparations. * denotes *p* < 0.05.

To examine how estrogens affect the association of Hsp70-1 and Hsc70 with chromatin, we cultured MCF7 cells under hormone-starvation conditions for 4 days and then treated the cells with either 100 nM E2 or ethanol (control) for 24 h, fractionated the treated cells into cytoplasmic (C), nuclear soluble (NS), transcriptionally active chromatin (Ch1) and inactive chromatin (Ch2) fractions, and analyzed those fractions with Western blotting. The E2 treatment caused significant reduction of ERα as a cytoplasmic protein and as a nuclear soluble protein, suggesting that E2 treatment causes translocation of ERα from the cytoplasm to the nucleoplasm, and eventually the majority of the soluble nuclear ERα to chromatin (Fig 6). In addition, E2 significantly increased the distribution of Hsp90α in the nucleus as nuclear soluble protein. Compared with the dynamic changes in ERα and Hsp90α, E2 had no significant effect on the distribution of Hsp70-1 and Hsc70 among the different fractions (Fig 6).

## Discussion

Through a quantitative proteomic approach, we identified 21 Hsps and 3 Hsp cochaperones that associate with ERα. The most abundant Hsps that were identified to associate with ERα were four Hsp70 members, followed by two Hsp90 members and three Hsp110 members when 293T cells were cultured in “complete” medium. Within the Hsp70 family, Hsp70-1 and Hsc70 stood out as the most abundant Hsps that associate with ERα, followed by Grp75 that is localized in the mitochondria, and Grp78 that is localized in the endoplasmic reticulum. The two most common Hsp90 family members, Hsp90α and Hsp90β [53], were also identified to abundantly associate with ERα, though at much less abundant levels than the four Hsp70 family members. It is generally believed that ERα interacts with Hsp90 only in the absence of ligands, and dissociates from Hsp90 in the presence of ligands [1, 33, 57]. In this study, although we did not add any exogenous estrogenic ligands (such as E2) to the media for culturing the 293T cells for proteomic identification, we cultured the cells in “complete” medium that contains phenol red, which is known to act as a weak estrogen to stimulate proliferation of ERα-positive cells [66] and FBS, which contains steroid hormones [67]. In addition, we included 10 nM E2 in the lysis buffer for preparing total cellular protein for LC-MS/MS analysis. The identification of Hsp90α and Hsp90β as ERα interacting proteins under the present cell culture and affinity purification conditions suggests that Hsp90 could also complex with ERα, at least partially, in the presence of estrogenic ligands. This conclusion is consistent with the notion that the dynamic and transient interaction of steroid-bound SRs with Hsp90 may be required for the cytoplasmic-nuclear trafficking of SRs in cells [61].

Historically, the attention in studying the role of Hsps in regulating the assembly, trafficking, and transcriptional activity of ERα has been focused on Hsp90 [1, 33]. Through conventional liquid chromatography or affinity purification, it has been well established that Hsp90 interacts with ERα in a variety of tissue/cells in the absence of ligands [33]. Because of its role in controlling SRs including ERs, and a separate role in protecting oncoproteins, Hsp90 inhibitors are in clinical trials for treating cancer [23, 24]. Compared with Hsp90, much less is known about Hsp70 in regulating ERα. In this study, we found that Hsp70-1 and Hsc70 were the most abundant Hsps that associate with ERα (Table 1). Interestingly, despite that the majority of Hsp70-1 and Hsc70 were localized in the cytoplasm, comparable amounts of cytoplasmic and nuclear hsp70-1 and Hsc70 were precipitated by anti-ERα antibody (Fig 2). Furthermore, significant portions of Hsp70-1 and Hsc70 were associated with active chromatin and inactive chromatin (Fig 3), and the two Hsps interacted with ERα in both forms of the chromatins (Fig 4). These results are consistent with the observation that the association of Hsp70 with SRs does not affect DNA binding activity of SRs [68]. In contrast, Hsp90α was almost exclusively localized in the cytoplasm and in the nucleus as non-chromatin-binding protein (Fig 3), which is consistent with the previous observations that SR-Hsp90 complexes are not associated with DNA and that dissociation of Hsp90 from SRs leads to DNA-binding of SRs [69, 70]. Unlike the ERα-Hsp90 association that is normally hormone-dependent [1, 33, 57], Hsp70 is still associated with SRs in the presence of steroid hormones [64, 65, 68, 71], which was also observed in this study (Fig 5). These results suggest that Hsp70 may play a dramatically different role in regulating ER biological activities compared with Hsp90. Perhaps, cells have evolved two distinct Hsp chaperone systems as repressors to keep ERα in the inactive states in transcription–one is “off-site” (not associated with chromatin) and ligand responsive, which is mediated by Hsp90, and one is “on-site” (associated with chromatin) and not/partially ligand responsive, which is mediated by Hsp70. If this is the case, it would be interesting to examine how these two chaperone systems interplay to regulate ERα transcriptional activities in a broad context such as tissue development and homeostasis.

In addition to functioning as nuclear receptors and transcription factors in the nucleus, ERs also act as signaling molecules in the plasma membrane and are localized in the mitochondria and the endoplasmic reticulum [72–74]. In this study, three mitochondrial Hsp members, Grp75, HSPE1, DNAJA3, were identified to associate with ERα. In particular, Grp75 was identified as a major ERα interactant (Table 1). When nuclear-gene-encoded proteins, such as ERα, are transported into mitochondria via posttranslational import, the proteins are imported into mitochondria in the unfolded states and need to be properly folded after the import. It would be interesting to determine whether Grp75, HSPE1, and DNAJA3 are merely responsible for folding imported ERα in the mitochondria or play additional roles in regulating ERα biological activities in the mitochondria. Several lines of evidence suggest that ERs may play important roles in the mitochondria. For example, it is known that a portion of cellular ERs are localized to mitochondria and the relative distribution of ERs into the mitochondrial pool is regulated by estrogens [47, 48, 75–78]. In addition, it has been shown that mitochondrial DNA contains estrogen response elements [79] and that mitochondrial structure and some important functions are influenced by estrogenic ligands. In addition to the mitochondria, ERs are also localized in the plasma membrane and the endoplasmic reticulum in the extra-nuclear compartments [72, 74, 80–82]. Posttranslational palmitoylation of ER can contribute to ER membrane localization [83]. Interestingly, both ERα and ERβ were found to localize in the rough endoplasmic reticulum and secretory vesicles [82]. This raises the possibility that ERs could also be transported to the membranes through vesicular transport. However, virtually nothing is known about endoplasmic reticulum ER besides its localization. In this study, we found that Grp78, an Hsp that is localized in the endoplasmic reticulum, was abundantly associated with ERα (Table 1). At present, it is not clear whether the identification of Grp78 as a potential ERα interactant reflects a need of this Hsp in mediating ERα in this organelle.

## Supporting Information

S1 Fig Comparison of extraction of inactive chromatin with 600 mM NaCl and sonication. There was an inconsistency between Figs 3 and 4 in the main text with regard to the relative content of ERα in inactive chromatin (Ch2). When inactive chromatin was extracted with 600 mM NaCl, which was the case for Fig 3, ERα content in inactive chromatin was the highest among the five fractions examined (Fig 3). However, when inactive chromatin was extracted with sonication, which was the case for Fig 4, ERα content was lower in inactive chromatin than in active chromatin (Fig 4, top panel). To examine whether the inconsistency was caused by different extraction methods, we extracted cytoplasmic (C), nuclear soluble (NS), and active chromatin (Ch1) from two populations of MCF7 cells as described in the main text, followed by extraction of inactive chromatin from the first population of cells with 600 mM NaCl and from the second population of cells with sonication. The results demonstrate that sonication extracted less ERα in inactive chromatin fraction compared to 600 mM NaCl extraction (S1 Fig), suggesting that the lower input ERα content in inactive chromatin fraction shown in the Fig 4 resulted from less efficient extraction of inactive chromatin by sonication compared to 600 mM NaCl extraction.
(TIFF)

S1 Table The list of peptides identified for the heat shock proteins and cochaperones that associate with estrogen receptor alpha. (XLS)
