# Supplementary figures and images for: Systematic Proteomic Identification of the Heat Shock Proteins (Hsp) that Interact with Estrogen Receptor Alpha (ERα) and Biochemical Characterization of the ERα-Hsp70 Interaction
Source: PLoS One. 2016 Aug 2;11(8):e0160312. doi: 10.1371/journal.pone.0160312 (PMC4970746; doi:10.1371/journal.pone.0160312)

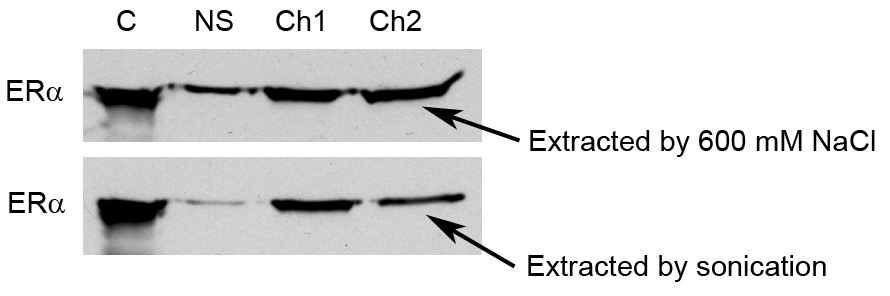

Supplement: S1 Fig — There was an inconsistency between Figs 3 and 4 in the main text with regard to the relative content of ERα in inactive chromatin (Ch2). When inactive chromatin was extracted with 600 mM NaCl, which was the case for Fig 3, ERα content in inactive chromatin was the highest among the five fractions examined (Fig 3). However, when inactive chromatin was extracted with sonication, which was the case for Fig 4, ERα content was lower in inactive chromatin than in active chromatin (Fig 4, top panel). To examine whether the inconsistency was caused by different extraction methods, we extracted cytoplasmic (C), nuclear soluble (NS), and active chromatin (Ch1) from two populations of MCF7 cells as described in the main text, followed by extraction of inactive chromatin from the first population of cells with 600 mM NaCl and from the second population of cells with sonication. The results demonstrate that sonication extracted less ERα in inactive chromatin fraction compared to 600 mM NaCl extraction (S1 Fig), suggesting that the lower input ERα content in inactive chromatin fraction shown in the Fig 4 resulted from less efficient extraction of inactive chromatin by sonication compared to 600 mM NaCl extraction. (TIFF) [file pone.0160312.s001.tiff]
